# Letter from a Foreign Correspondent

**Published:** 1847-09

**Authors:** 


ART. IV.—*Letter from a Foreign Correspondent.*—The following letter giving some account of medical matters at Liverpool, England, is furnished by a correspondent who prefers to withhold his signature. The writer is well known by the Editor, and we trust that the account of his observations may possess interest for our readers.—EDITOR BUFFALO MEDICAL JOURNAL.

In Liverpool there are thirteen Medical charities, of which we have visited the Lock Hospital, the Northern Dispensaŋy, North Leeds' Hospital, and the Liverpool Infirmary. This last, the Liverpool Infirmary, near the London road, is the most extensive in the city, having 230 beds. On one of the operating days we were invited by Mr. Bickersteth to attend in the operating theatre. There were about twenty students present, with two of the surgeons to the establishment, viz:—Mr. Bickersteth and Mr. Halton. It is also, I think, the oldest and is tolerably well endowed; but it receives its chief support from an annual subscription made up of amounts varying generally from a few shillings to $40 or $50. The whole amount thus received annually is about $15,000. Three amputations were made for scrofulous enlargements of the knee joint; all of them amputated above the knee, by the circular mode. I am certain that in the Massashusetts Hospital, not one of these limbs would have been condemned. But this is the practice generally adopted in this establishment, to amputate at an early stage of the disease. By this practice they affirm that they save the patient many months or years of suffering, and the disease does not, or *has* not in a single instance developed itself in the lungs or other parts of the body, It is a practice, however, so contrary to the conservative plan of American surgeons, and so contrary to my establish prejudices that you will not, as a matter of course expect me to sanction it. I honestly believe that with the present improved treatment of such cases, by iodine in some of its forms, and *complete rest*, every one of those limbs might have been saved. Yet it is doubtless true, that if the limb cannot be saved, the sooner it is cut off the better; and it never ought to be removed when hectic is fairly established.

So far as the operation is concerned, I have never seen it done better. Two of the amputations were made by Mr. Halton, and one by Mr. Bickersteth.

The Lunatic Asylum, under the care of Mr. Tyrrell, is near the Infirmary, and most beautifully situated. All its arrangements are most perfect and systematic. No coercion is ever used, and a straight jacket was never seen in the establishment. Among many other precautionary measures, we noticed particularly german silver forks; knives with dull edges, except about two inches near the end; strong grates enclosing the fires, and locked up. Mr. Formby is the physician to the Asylum. Mr. F. informs me that the average weekly number of patient is about 60. During the last year 194 have been received, of whom 55 have been cured.

The Lock Hospital, which, with the Asylum and Infirmary, is under the same general board of managers, has now about fifty patients, and has received during the last year over 400, of whom says Dr. Worthington, one of the surgeons, *only one has died*. This is a success which speaks well for the medical attendants, since the mass of the patients are furnished by the trading vessels, and as most of these sailing from Liverpool make long voyages, the patients are often brought in, in a very much diseased condition. The treatment pursued here is eclectic, mercury is employed, but with care and discrimination.

The Liverpool Northern Hospital is badly located, but a new building is soon to be erected on a more healthy site. When we visited it, we found no one but the House Surgeon, Mr. Parker, at home. The institution has three surgeons and two physicians, besides a consulting surgeon. To the office of surgeon no man is eligible, who has not taken his diploma at either the college of surgeons in London, Dublin, Edinburg or Glasgow. The same rule is adopted in nearly all, if not all of the English Hospitals and Dispensaries. I believe all chartered medical charities are required by law to adopt the same rule.

You see that the diplomas of two Scotch schools are received, and but one English and one Irish. Liverpool has a Medical School, with about twenty students, and it can never have many more, notwithstanding the Professors may have equal ability, and the city furnishes equal facilities for instruction, until this law is repealed. It may be well enough, but it is not quite as democratic as the laws at home.

By a similar law, no Physician can hold office in this charity who has not taken his degree in the arts, either at the University of Oxford or Cambridge, of E[illegible]nburgh or Glasgow, or of Dublin. Nor can he be elected as Phys[illegible]an, if he practice Surgery, Midwifery or Pharmacy.

The annual income of this charity is about $15,000. The major part of which also is raised by small private subscriptions.

There are also in the city two excellent Dispensaries, the North and South Dispensary, sustained almost entirely by annual donations and subscriptions. I allude to the manner in which these charities are sustained, because it struck me as singular that in a city with a population of nearly or quite 300,000, and in which there is so much wealth, so little permanent provision should be made for the indigent sick. Every city of the ability of Liverpool, ought to provide ample Hospital and Dispensary accommodations for its poor: and especially is this city bound to make such provision, since, according to Mr. Duncans rates of mortality, this is the most unhealthy city in England, and the corporation owns property valued at not less than $15,000,000, which it can expend for any public purpose, and some of which has been appropriated even to the building of churches and *private* burying grounds. When I speak of "private" burying grounds, I refer to the beautiful cemetery of St, James, which was originally a redstone quarry within the limits of the city and on its hightest ground. This place I visited yesterday with J., and I will explain my reference. It is an excavation of a crescentic form, about forty feet deep, and about 100 rods long by 20 rods wide, which was purchased by the corporation, enclosed with an iron fence, and elegantly laid out for the accommodation of the dead. For eighteen guineas you may here purchase a lot of seven feet by three, already excavated and walled up with brick, and in a most respectable neighborhood; but for seventeen guineas more, you may have a residence in one of the catacombs, of which there are many, excavated from the solid rock along the sides of the cemetery, and occupied chiefly by the aristocracy: and if you have money left, you may purchase the right of erecting a marble statue to your memory in the beautiful little stone chapel near the main entrance. But if you have no money you cannot rot here—there are noses near which the scent of such poor clay would offend; or, though your purse be full of gold, if, of the ninety-one christain churches in Liverpool, you have prayed in either of the fifty-nine dissenting churches—if you are a Baptist, or a Presbyterian, or a Methodist, or any thing else than a member of one of the thirty two "established churches," you might jingle your gold against the bars of this enclosure until the resurection day, and never would its gates be opened for you—yet this cemetery is built and adorned with the public money! By what right are the majority, the most honest as well as the poorest taxed, to furnish sepulture for the minority, the least honest and the richest? Shame upon such a government, where "the church" is the constant offence to the people, and by which even the Hospitals and other public charities are forever defrauded of their just revenues.

The Park, adjoining the cemetery, is a delightful spot for an evenings' walk, but the trees, the benches, and indeed every thing else in Liverpool, is black with soot. The shrubbery in the Park has such a black, gnarled and unhealthy look, that I could scarcely recognize some of our most familiar plants and trees. The air is constantly loaded with coal smoke, and must prove I think as poisonous to human as it evidently does to vegetable life; and to this, quite as much as to the narrow and crowded streets, must be attributed the low rate of life in this city.

I say I have omitted one of the most active causes of mortality in this city—"The Gin Palaces"—or "Hells"—which abound to an almost incredible extent. In several of the principal streets their average number, compared with other buildings, is as one to every three or four, and a considerable proportion of their customers are women. They are frequented all hours of the day, but are *thronged* from seven o'clock to ten or eleven at night. But I must close this very long letter, which has already consumed too much of my valuable ship paper, and too much of your valuable time in the reading.

